# Influence of Nd Substitution on the Phase Constitution in (Zr,Ce)Fe_10_Si_2_ Alloys with the ThMn_12_ Structure

**DOI:** 10.3390/ma16041522

**Published:** 2023-02-11

**Authors:** Mieszko Kołodziej, Jean-Marc Grenèche, Sandy Auguste, Bogdan Idzikowski, Maciej Zubko, Lotfi Bessais, Zbigniew Śniadecki

**Affiliations:** 1 Institute of Molecular Physics, Polish Academy of Sciences, Mariana Smoluchowskiego 17, 60-179 Poznań, Poland; 2 NanoBioMedical Centre, Adam Mickiewicz University, Wszechnicy Piastowskiej 3, 61-614 Poznań, Poland; 3 Institut des Molécules et Matériaux du Mans (IMMM, UMR CNRS 6283), Le Mans University, Avenue Olivier Messiaen, CEDEX 09, 72085 Le Mans, France; 4 Institute of Materials Engineering, Faculty of Science and Technology, University of Silesia, 75 Pułku Piechoty 1a, 41-500 Chorzów, Poland; 5 Department of Physics, Faculty of Science, University of Hradec Králové, Rokitanského 62, 500 03 Hradec Králové, Czech Republic; 6 CNRS, ICMPE, University Paris Est Creteil, UMR 7182, 2 Rue Henri Dunant, 94320 Thiais, France

**Keywords:** ThMn_12_-type phase, permanent magnet, Mössbauer spectrometry, crystalline structure

## Abstract

Iron-based compounds with a ThMn_12_-type structure have the potential to bridge the gap between ferrites and high performance Nd_2_Fe_14_B magnets. From the point of view of possible applications, the main advantage is their composition, with about 10 wt.% less rare earth elements in comparison with the 2:14:1 phase. On the other hand, the main issue delaying the development of Fe-rich alloys with a ThMn_12_-type structure is their structural stability. Therefore, various synthesis methods and stabilizing elements have been proposed to stabilize the structure. In this work, the influence of increasing Nd substitution on the phase constitution of Zr_0.4−x_Nd_x_Ce_0.6_Fe_10_Si_2_ (0 ≤ x ≤ 0.3) alloys was analyzed. X-ray diffraction and ^57^Fe Mössbauer spectrometry were used as the main methods to derive the stability range and destabilization routes of the 1:12 structure. For the arc-melted samples, an increase in the lattice parameters of the ThMn_12_-type structure was observed with the simultaneous growth of bcc-(Fe,Si) content with increasing Nd substitution. After isothermal annealing, the ThMn_12_-type structure (and the coexisting bcc-(Fe,Si)) were stable over the whole composition range. While the formation of a 1:12 phase was totally suppressed in the as-cast state for x = 0.3, further heat treatment resulted in the growth of about 45% of the ThMn_12_-type phase. The results confirmed that the stability range of ThMn_12_-type structure in the Nd-containing alloys was well improved by other substitutions and the heat treatment, which in turn, is also needed to homogenize the ThMn_12_-type phase. After further characterization of the magnetic properties and optimization of microstructure, such hard/soft magnetic composites can show their potential by exploiting the exchange spring mechanism.

## 1. Introduction

Currently, permanent magnets are widely used and the most known among them, with the highest energy product *BH*_|max|_, are based on rare earth elements (REE), such as Sm or Nd [1,2], with well-known representatives in Nd_2_Fe_14_B [3], Sm_2_Co_17_ [4], and Sm_2_Fe_17_N_3_ [5]. The range of possible applications is broad and is still growing [6]. With the development of new phases and the improvement of intrinsic and extrinsic properties, permanent magnets are still implemented in a rather trivial way, as a replacement of electromagnets, in loudspeakers and actuators, but also find more sophisticated applications in magnetic resonance imaging, in information storage devices, magnetic levitation, as bearings, or as miniaturized magnetic field sensors [6,7]. The demand is still growing, as huge quantities of permanent magnets are used in, e.g., the automotive industry or wind turbine production [8]. Their criticality (growing demand with unstable supply chains) has led to the extensive research of new materials [9], where two important directions can be distinguished: (i) the decrease of REE content with preservation of magnets’ magnetic performance and (ii) the search for novel REE-free hard magnetic materials, e.g., L1_0_-FeNi [10] known from meteorites [11]. Moreover, there is a renewed interest in the tetragonal ThMn_12_-type (1:12 stoichiometry) phase, which has already been known for decades [12]. This tetragonal structure is related to that of SmCo_5_ of CaCu_5_-type, where half of the rare earth atoms are replaced with transition metal dumbbells [7]. Even if its *BH*_|max|_ would be slightly lower compared with high performance magnets, it would be still placed in the performance gap between cheaper ferrite-based and more expensive Nd-based magnets with 2:14:1 structure [13]. Nevertheless, another issue that has to be solved with these compounds is their structural stability. For most Fe- and REE-based compounds, ThMn_12_-type phase is less stable than, for example, the hexagonal 2:17-type structure [14]. Therefore, stabilizing elements, such as Ti, V, Si or Al, have to be used [15,16,17]. For example, the SmFe_12_ compound forms as a thin film, but is unstable in bulk [18]. In such cases, large transition metal atoms are needed to stabilize the structure, as in SmFe_11_Ti [19]. The simultaneous substitution of other elements, for example, Zr on rare earth sites and Co on Fe sites, allowed a decrease in Ti content in (Sm_0.8_Zr_0.2_) (Fe_0.75_Co_0.25_)_11.5_Ti_0.5_ and the optimization of the magnetic properties [20]. Additionally, there are several further issues that must be taken into account, such as microstructure and twinning [18,21]. One of the largest groups of alloys with the ThMn_12_ structure is based on Sm [22,23,24,25]. Nonetheless, for already known and investigated 1:12-type compounds, the maximum effort was made to develop Ce-based compounds due to the low criticality of this element [26,27,28]. Recently, the formation of the ThMn_12_ structure (almost 100% of volume fraction) has been confirmed in Zr_0.4_Ce_0.6_Fe_10_Si_2_ [29]. The Ce substitution increased the anisotropy field from 16.9 kOe for ZrFe_10_Si_2_ to 24 kOe for Zr_0.4_Ce_0.6_Fe_10_Si_2_. Replacement of Zr by Nd in ZrFe_10_Si_2_ and further nitrogenation of this alloy have also been investigated [30] and are believed to be very promising in the optimization of hard magnetic properties. Nd_0.4_Zr_0.6_Fe_10-_Si_2_ has been also examined to confirm whether the formation of the ThMn_12_-type phase is possible and to determine its magnetic and structural properties [31]. It has also been shown than the replacement of Fe atoms by Co improved magnetic performance, with the enhancement of the Curie temperature as a main benefit [31]. These results raised a question: how would substitution of Zr by Nd in Zr_0.4_Ce_0.6_Fe_10_Si_2_ affect formation of the 1:12 structure [29]? Nd is believed to enhance the magnetic performance of this alloy [32]. As-cast samples were also isothermally annealed at 1373 K, the temperature which has been reported before [33], to lie in the stability region for ThMn_12_ phase formation. Therefore, on the basis of structural and spectroscopic measurements, the structural stability of the ThMn_12_-type phase in the Nd-substituted Zr_0.4_Ce_0.6_Fe_10_Si_2_ alloy was determined in this paper.

## 2. Methods

Samples with Zr_0.4−x_Nd_x_Ce_0.6_Fe_10_Si_2_ (0 ≤ x ≤ 0.3) compositions were prepared by arc-melting in an argon atmosphere. The melting procedure was repeated several times to ensure homogeneity. Additionally, residual oxygen was gathered by melting a Zr getter. Afterwards, samples wrapped in Ta foil were placed in quartz capsules, which were then evacuated three times with the use of turbomolecular pump (down to 10^−4^ mbar), refilled with Ar up to 500 mbar and sealed. Samples were then annealed isothermally at 1373 K in the Carbolite MTF 12/25/250 tube furnace for 72 h and subsequently quenched in cold water. For microscopic measurements, the powdered sample was suspended in isopropyl alcohol, and the resulting material, after dispersion in an ultrasonic bath for 30 min, was deposited on a Cu grid with an amorphous carbon film standardized for transmission electron microscope (TEM) observations. Structural analysis was performed using X-ray powder diffraction (XRPD). Patterns were recorded with a PANalytical X’Pert Pro diffractometer in Bragg–Brentano geometry (CoK_α_ radiation). A cobalt anode was chosen to avoid X-ray fluorescence of iron compounds. Data were collected in the range of 2*θ* from 20 to 110° with a 0.0167° step and 400 s per step for a total acquisition time of 5 h. X-ray diffraction patterns were analyzed using MAUD software (Rietveld method combined with Fourier analysis) [34] to determine the phase constitution, volume fractions and lattice parameters of various phases. Transmission ^57^Fe Mössbauer effect experiments were performed at room temperature (RT) using a constant acceleration conventional spectrometer with a ^57^Co source. Samples were powdered using an Fe-free tool. The powder obtained was collected and placed in an altuglass sample holder with a content of about 5 mg Fe/cm^2^. The hyperfine structure was modeled by least square refinement involving magnetic and quadrupolar components with Lorentzian lines. The values of the isomer shift are quoted relative to those of bcc-Fe at RT. Initial measurements were made in the range from −12 to 12 mm/s, to check the presence of oxides. Final spectra with improved statistics were measured from −8 to 8 mm/s. Microstructure analysis of the selected samples was carried out using JEOL JEM-3010 high-resolution transmission electron microscope with 300 kV acceleration voltage, equipped with a Gatan 2k × 2k Orius™ 833 SC200D CCD camera. Recorded selected area electron diffraction (SAED) patterns, as well as the calculated Fast Fourier Transforms (FFT) from high-resolution images were indexed using ElDyf [35] computer software.

## 3. Results and Discussion

**Figure 1:**
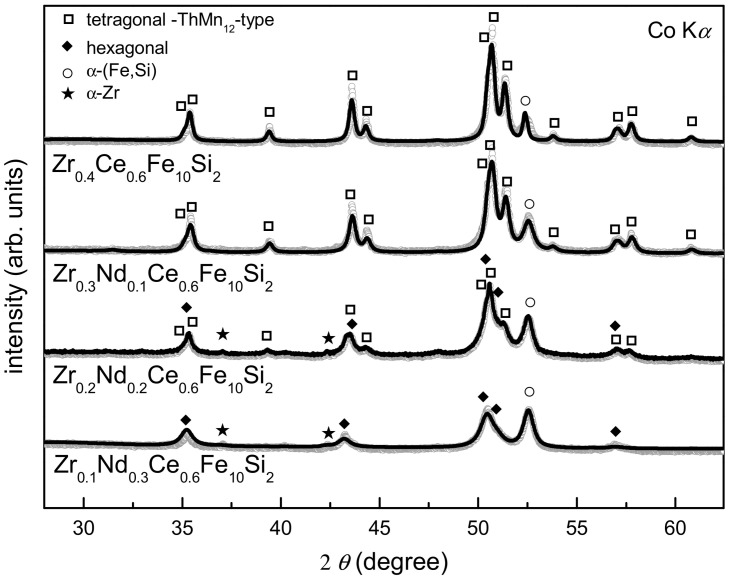
X-ray diffraction patterns of arc-melted Zr_0.4−x_Nd_x_Ce_0.6_Fe_10_Si_2_ (0 ≤ x ≤ 0.3) alloys. ThMn_12_-type structure, α-(Fe,Si), α-Zr and highly disordered hexagonal structure were identified and marked with respective symbols.

**Figure 2:**
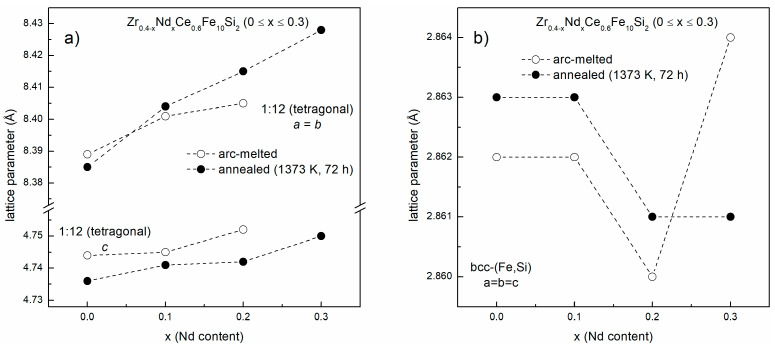
Refined values of lattice parameters for (**a**) ThMn_12_ and (**b**) bcc-(Fe,Si) structures according to Nd content for arc-melted (white points) and annealed at 1373 K for 72 h (black points) Zr_0.4−x_Nd_x_Ce_0.6_Fe_10_Si_2_ (0 ≤ x ≤ 0.3) alloys.

The X-ray diffraction patterns for Zr_0.4−x_Nd_x_Ce_0.6_Fe_10_Si_2_ (0 ≤ x ≤ 0.3) alloys synthesized by the arc-melting method are shown in Figure 1. The Zr_0.4_Ce_0.6_Fe_10_Si_2_ alloy contains about 90% of the ThMn_12_-type structure with the addition of about 10% of bcc-(Fe,Si) phase. The presence of Si in bcc structure was expected, taking into account changed lattice parameter values (Table 1), and was confirmed by spectroscopic measurements. By replacing 25 at.%, 50 at.% and 75 at.% of Zr by Nd, we obtained nominally Zr_0.3_Nd_0.1_Ce_0.6_Fe_10_Si_2,_ Zr_0.2_Nd_0.2_Ce_0.6_Fe_10_Si_2_, and Zr_0.1_Nd_0.3_Ce_0.6_Fe_10_Si_2_ alloys. In the first case, about 82% of the ThMn_12-_type structure and 18% of bcc-(Fe,Si) phase formed. Substitution of 50% of Zr by Nd led to further suppression of ThMn_12_-type phase formation and facilitated the growth of highly disordered hexagonal phase, nominally (REE)Fe_5_. About 52% of ThMn_12_-type phase formed along with 25% of the bcc-(Fe,Si) phase and an insignificant amount of α-Zr. One must bear in mind that the fitting procedure for an α-Zr phase is based just on two reflections. Therefore, this finding should be treated with a caution. Nevertheless, for clarity reasons, we use this designation throughout the paper. In Zr_0.1_Nd_0.3_Ce_0.6_Fe_10_Si_2_, the ThMn_12_-type structure was not observed, while the presence of hexagonal-type phase, bcc-(Fe,Si) phase and α-Zr was confirmed. The bcc-(Fe,Si) lattice parameter does not vary much depending on the Nd content, and the bcc lattice contained about 8–10 at.% of Si in every investigated sample. The Nd influence on the values of ThMn_12_-type phase lattice parameters and lattice volume was noticed, where all parameters (a = b and c) increased with the growth of Nd content (Table 1). Along with the influence of thermodynamic parameters (e.g., enthalpies of formation), lattice expansion could be one of the main reasons for the instability of the ThMn_12_-type structure for large substitutions of Zr by Nd. Detailed results are presented in Figure 2. Zirconium and silicon play a significant role in the stabilization of the ThMn_12_-type structure in Fe-based alloys [29,30]. The influence of Nd, Ce and Zr on the enthalpies of formation of various phases in the Fe-Si-based alloys has been also analyzed by us lately [36]. Beneficial influence of Zr on the formation of the ThMn_12_-type phase was confirmed, but the enthalpy of formation of other competing phases (i.e., bcc solid solution and amorphous phase) also decreased significantly with increased Zr content. When we analyzed enthalpy values only, it has been reported that Ce and Nd should have deteriorating impact on the stability of crystalline phases. Nevertheless, with small rare earth element substitutions, other quantities such as a mismatch entropy [36] or site preference [30] play a role in the formation of ThMn_12_-type phase.

**Figure 3:**
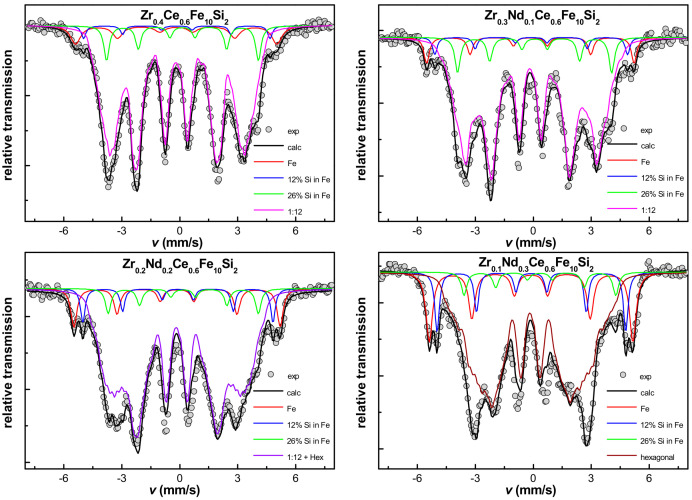
Mössbauer spectra in arc-melted Zr_0.4−x_Nd_x_Ce_0.6_Fe_10_Si_2_ (0 ≤ x ≤ 0.3) alloys.

Mössbauer spectrometry was used to support the structural investigations. Several sextets with different hyperfine field parameters, suggesting a multiphase character of investigated samples and numerous magnetically distinguishable surroundings of Fe atoms were observed. As already mentioned, the spectrum initially assigned in the fitting procedure to be bcc-Fe was non-typical, as it was composed of at least three sub-spectra. The determined hyperfine parameters values were confronted with the literature data [37]. We considered the values of specific hyperfine field parameters and applied them in a fitting model according to wt.% of Si in Fe [37]. The results suggested, along with the lattice parameter value of bcc phase, that the observed bcc-Fe phase was in fact the bcc-(Fe,Si). Part of Si diffuses into the bcc lattice and is not directly involved in the ThMn_12_-type structure stabilization. Mössbauer spectra are shown in Figure 3, while hyperfine parameters are listed in Table 2. Notably, more precise estimation of Si content in bcc lattice was possible, as the lattice parameter of bcc structure and Si content were linearly dependent on each other [38]. Therefore, it was possible to extrapolate refined lattice parameter to confirm the content of Si diffusing into the bcc structure.

**Figure 4:**
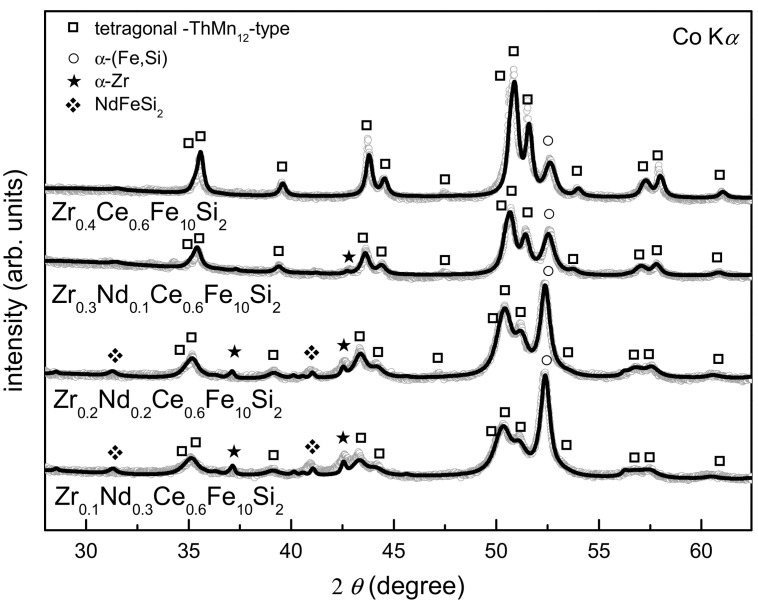
X-ray diffraction patterns of Zr_0.4−x_Nd_x_Ce_0.6_Fe_10_Si_2_ (0 ≤ x ≤ 0.3) alloys annealed at 1373 K for 72 h, mainly composed of ThMn_12_-type structure.

Afterwards, isothermal annealing of arc-melted alloys at 1373 K for 72 h (conditions favorable for ThMn_12_-type structure formation [33]) was performed. X-ray diffraction patterns are shown in Figure 4. After heat treatment, higher precipitation of bcc-(Fe,Si) was observed for the parent Zr_0.4_Ce_0.6_Fe_10_Si_2_ alloy, with about 17% of total volume fraction, when compared to 10% in the arc-melted sample. A small amount of 2:17 structure formation and an insignificant amount of ZrFeSi_2_ phase were also noticed. In contrast to as-cast samples, the ThMn_12_-type phase was present in the whole composition range in the annealed samples. As observed in arc-melted samples already, Nd-substitution led to a decreasing volume fraction of the ThMn_12_-type structure. The inverse dependence was observed for the bcc-(Fe,Si) phase. The amount of hexagonal 2:17-type phase and ZrFeSi_2_ phase did not exceed 5 at.% in the whole composition range. Silicon content in the bcc lattice was determined by estimating the lattice parameter [37,38]. Its content is in the range of 9–10 at.%, in accordance with the previous results for the as-cast samples. Lattice parameters of ThMn_12_-type phase increased with Nd content (Table 1). As the refinement of the lattice parameters was made using the Rietveld method, the estimated standard deviation was the only measure of the uncertainty of the lattice parameters which did not exceed 0.001 Å.

**Figure 5:**
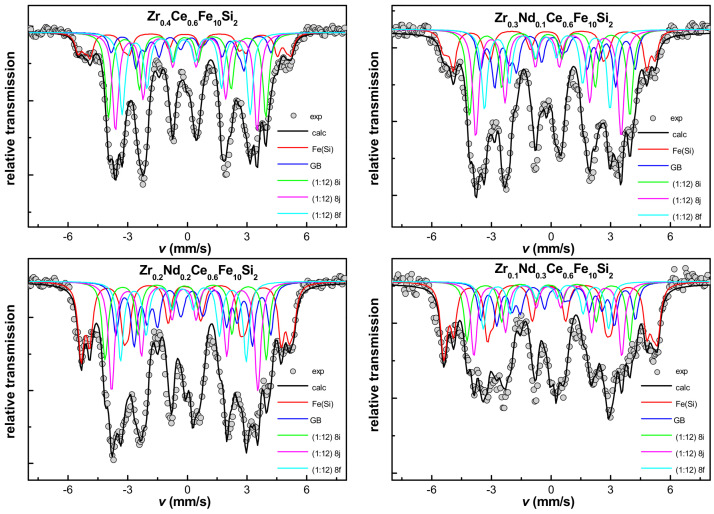
Mössbauer spectra of Zr_0.4−x_Nd_x_Ce_0.6_Fe_10_Si_2_ (0 ≤ x ≤ 0.3) alloys annealed at 1373 K for 72 h.

The Mössbauer spectrometry results showed that the ThMn_12_-type structure obtained after arc-melting was clearly not homogeneous. As it was not possible to describe three subspectra for 8i, 8j and 8f positions (Figure 3), a distribution of parameters was used by similarity to these already known from previous investigations, where Ce substitution for Nd in similar alloys was reported to be responsible for lowering of the hyperfine field (*B*_hf_) [39,40,41]. Consequently, the mean value of the hyperfine field parameter was checked and estimated to be equal to 28.7 ± 0.5, 28.7 ± 0.5, 29.9 ± 0.5 and 30.6 ± 0.5 T for the Zr_0.4_Ce_0.6_Fe_10_Si_2_, Zr_0.3_Nd_0.1_Ce_0.6_Fe_10_Si_2_, Zr_0.2_Nd_0.2_Ce_0.6_Fe_10_Si_2_, and Zr_0.1_Nd_0.3_Ce_0.6_Fe_10_Si_2_, respectively. This slight increase was connected with the growth of a disordered hexagonal phase replacing the ThMn_12_-type one. All hyperfine parameters determined from the spectra shown in Figure 3 are presented in Table 2. We observed the evolution of spectra with Nd substitution, which is in accordance with X-ray diffraction results, and we concluded that the content of pure bcc-Fe phase (with no Si in the nearest neighborhood) increases from 3% for Zr_0.4_Ce_0.6_Fe_10_Si_2_, to 6, 11 and 17% for Zr_0.3_Nd_0.1_Ce_0.6_Fe_10_Si_2_, Zr_0.2_Nd_0.2_Ce_0.6_Fe_10_Si_2_, and Zr_0.1_Nd_0.3_Ce_0.6_Fe_10_Si_2_, respectively. The same trend occurred for the bcc phase sub spectrum that was assumed to contain 12 at.% of Si (from 2, through 3, 7 up to 11% with increasing Nd content). The content of another sub-spectrum (bcc phase with 26 at.% of Si in Fe) is equal to about 6% for each sample. Small inaccuracies are possible because of overlapping spectra with hyperfine field parameters of about 24 T, coming from both the ThMn_12_-type structure and bcc phase with 26 at.% of Si in Fe. Annealed samples revealed much better homogeneity than arc-melted alloys, as shown by more consistent positions and narrower peaks observed in Mössbauer spectra (Figure 5). The spectra for 8i, 8j and 8f positions for the ThMn_12_-type phase were fully described. The hyperfine field parameter of Fe in 8i and 8j positions increased with the substitution of Nd for Zr (Table 3). Variation of *B*_hf_ was not so evident for the 8f position. It changed from 19.8 ± 0.5, through 19.4 ± 0.5, 19.5 ± 0.5 to 19.4 ± 0.5 T with the increasing Nd content. There is also a doublet visible in the middle of the Mössbauer spectra of all annealed samples, whose contribution increased with Nd substitution. It was assumed to originate from (Zr,Nd,Ce)FeSi_2_ phase, as its content also follows the annealed alloys’ constitution determined on the basis of X-ray diffraction. One must bear in mind the presence of grain boundaries (GB), which may play a large role while analyzing the Mössbauer spectra. Their contribution was estimated to be equal to about 10%, with a *B*_hf_ mean value varying from 19.8 ± 0.5 T for Zr_0.4_Ce_0.6_Fe_10_Si_2_, through 18.7 ± 0.5, 18.3 ± 0.5, down to 18.2 ± 0.5 T, for Zr_0.3_Nd_0.1_Ce_0.6_Fe_10_Si_2,_ Zr_0.2_Nd_0.2_Ce_0.6_Fe_10_Si_2_, and Zr_0.1_Nd_0.3_Ce_0.6_Fe_10_Si_2_, respectively. Their presence can have a great influence on the magnetic properties of nanocomposite [40,41].

**Figure 6:**
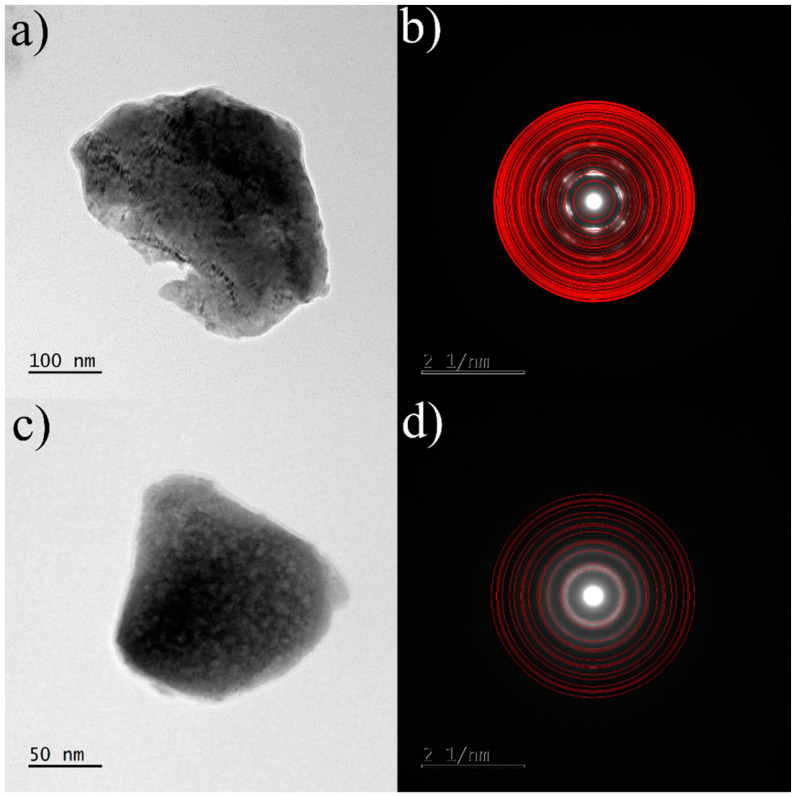
TEM images of the arc melted Zr_0.4_Ce_0.6_Fe_10_Si_2_ sample: (**a**) bright field image of the particle with predominantly ThMn_12_-type phase with (**b**) corresponding selected area electron diffraction pattern and (**c**) bright field image of the particle with predominantly bcc-(Fe,Si)-like phase with (**d**) corresponding selected area electron diffraction pattern. Red rings on the SAED patterns indicate theoretical Bragg positions for both phases.

**Figure 7:**
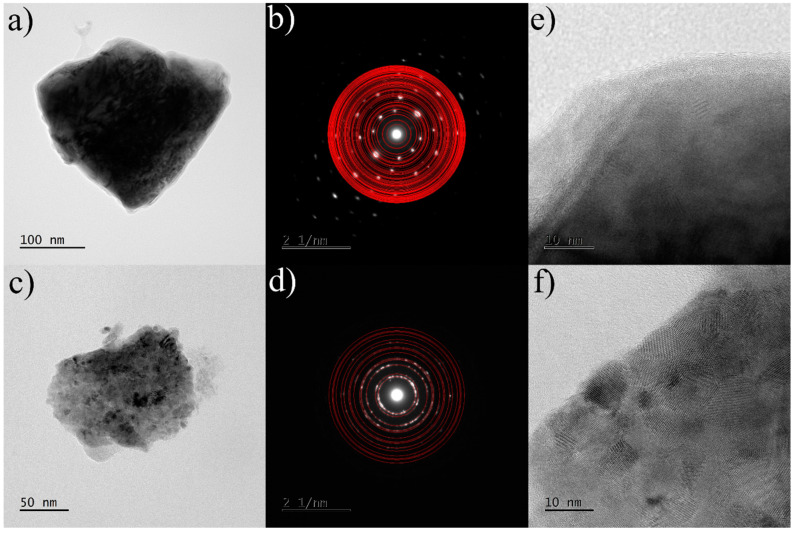
TEM images of the Zr_0.4_Ce_0.6_Fe_10_Si_2_ sample annealed at 1373 K for 72 h: (**a**) bright field image of the particle with predominantly ThMn_12_-type phase with (**b**) corresponding selected area electron diffraction pattern and (**c**) bright field image of the particle with predominantly bcc-(Fe,Si)-like phase with (**d**) corresponding selected area electron diffraction pattern. High-resolution images of the ThMn_12_-like phase and bcc-(Fe,Si)-like phase are shown in (**e**,**f**), respectively. Red rings on the SAED patterns indicate theoretical Bragg positions for both phases.

To examine the microstructure of the samples obtained and to confirm the phase composition obtained by X-ray diffraction, transmission electron microscopy (TEM) was performed on two selected samples: arc melted and annealed Zr_0.4_Ce_0.6_Fe_10_Si_2_. The TEM measurements were performed mainly to confirm the existence of grain boundaries, which were presumed mainly on the basis of Mössbauer spectrometry results. We took advantage of the use of this method to confirm simultaneously that, in fact, we deal mainly with ThMn_12_-type and (Fe,Si)-bcc phases. Nevertheless, we observed that the phase constitution and the mean crystallite size differ for various pieces of the same sample. Therefore, we omitted a more detailed analysis as it would not bring the results, which are representative for the sample as a whole. Nonetheless, the sample after arc-melting showed coarse grains. Electron diffraction measurements confirmed the presence of the tetragonal ThMn_12_-type structure and the bcc-(Fe,Si) phase (Figure 6). TEM observations of the annealed sample were in good agreement with the X-ray diffraction results. The tetragonal ThMn_12_-type phase and bcc-(Fe,Si) phase were also observed in the annealed sample. It was not able to determine the crystallite sizes of ThMn_12_-type phase precisely. For the bcc-(Fe,Si) phase, the recorded high-resolution images revealed a well-crystallized nanocrystalline structure with a crystallite size of about 9 nm (Figure 7). Some grain boundaries and/or structurally disordered regions are also visible, as suggested before by Mössbauer spectrometry.

## 4. Conclusions

Several Zr_0.4−x_Nd_x_Ce_0.6_Fe_10_Si_2_ (0 ≤ x ≤ 0.3) alloys mainly composed of a ThMn_12_-type structure were synthesized by arc-melting and subsequent annealing. The presence of the bcc phase, which was found to contain Si with a rather stable content throughout the whole series of samples (about 9–10 at.% of Si in bcc-Fe lattice), was confirmed for all samples. Moreover, a facilitated formation of bcc-(Fe,Si) was observed with increasing Nd content. The ThMn_12_-type structure became less stable with increasing Nd content, also due to a slight increase in lattice parameters/volume. The ^57^Fe Mössbauer spectrometry results confirmed that the ThMn_12_-type structure in the pre-annealed samples was highly disordered, and that the distribution of hyperfine parameters had to be used to describe the spectra correctly. For an equiatomic Zr/Nd composition, the crystallization of a highly disordered hexagonal phase, nominally (REE)Fe_5_, was observed. This process resulted in a simultaneous decrease in the driving force for the formation of a ThMn_12_-type structure (totally suppressed for x = 0.3). In contrast, the ThMn_12_-type phase was present in the whole composition range, along with the bcc-(Fe,Si), in the annealed samples. In addition, the growth of the 2:17 hexagonal phase was observed, whose content increased with Nd substitution (from 2 at.% up to 5 at.%). Importantly, the ThMn_12_ structure became more homogeneous after annealing. The homogeneity, as well as the improved stability, of the ThMn_12_-type phase (even for high Nd substitution) confirms that the preconceived synthesis route and the choice of stabilizing elements (Si, Zr, Nd, Ce) were appropriate for the future development of these alloys. It was evident that Nd adversely affected stability of 1:12 structure, but it was confirmed that the preservation of its high content was still possible (with Zr, Ce and Si substitution). Further experiments are needed to determine the influence of Nd-substitution on magnetic properties. Moreover, attention should be drawn to the possibilities of exploitation of the exchange spring effect, due to the presence of a soft magnetic bcc phase. By optimizing the content of hard and soft magnetic phases and their microstructure [42], one can expect improved values of coercivity and remanent magnetization, leading to the maximization of overall magnet performance.

## Figures and Tables

**Table 1:** Refined values of the lattice parameters of the two phases identified for Zr_0.4−x_Nd_x_Ce_0.6_Fe_10_Si_2_ (0 ≤ x ≤ 0.3) samples after arc-melting and annealing at 1373 K for 72 h: bcc-(Fe,Si) and ThMn_12_-type structure.

Nd Content		x = 0 Arc-Melted	x = 0 Annealed	x = 0.1 Arc-Melted	x = 0.1 Annealed	x = 0.2 Arc-Melted	x = 0.2 Annealed	x = 0.3 Arc-Melted	x = 0.3 Annealed
Phase	Lattice Parameter								
bcc-(Fe,Si)	a = b = c [±0.001]	2.862	2.863	2.862	2.863	2.860	2.861	2.864	2.861
ThMn_12_	a = b [±0.001]	8.389	8.385	8.401	8.404	8.405	8.415	-	8.428
	c [±0.001]	4.744	4.736	4.745	4.741	4.752	4.742	-	4.750

**Table 2:** Refined values of the hyperfine parameters of the phases identified for arc-melted Zr_0.4−x_Nd_x_Ce_0.6_Fe_10_Si_2_ (0 ≤ x ≤ 0.3) samples.

	Phase		B_hf_ (T) [±0.5]	δ (mm/s) [±0.01]	2ε (mm/s) [±0.01]	Area % [±2]
Zr_0.4_Ce_0.6_Fe_10_Si_2_						
	ThMn_12_		<28.7>	<−0.13>	<0.06>	88
	bcc-(Fe,Si)	bcc-Fe	32.3	−0.17	0	3
		Fe—12% Si	30.4	−0.1	0	2
		Fe—26% Si	24.5	−0.1	0	7
Zr_0.3_Nd_0.1_Ce_0.6_Fe_10_Si_2_						
	ThMn_12_		<28.7>	<0.05>	<0.05>	83
	bcc-(Fe,Si)	bcc-Fe	33.1	−0.15	0	6
		Fe—12% Si	30.8	−0.11	0	3
		Fe—26% Si	24.6	0.07	0	8
Zr_0.2_Nd_0.2_Ce_0.6_Fe_10_Si_2_						
	ThMn_12_ + hex		<29.9>	<−0.14>	<0.0>	76
	bcc-(Fe,Si)	bcc-Fe	32.8	−0.14	0	11
		Fe—12% Si	30.3	−0.09	0	7
		Fe—26% Si	24.1	0.18	0	6
Zr_0.1_Nd_0.3_Ce_0.6_Fe_10_Si_2_						
	Nd(Zr, Ce)Fe hex		<30.6>	<−0.14>	<0.02>	66
	bcc-(Fe,Si)	bcc-Fe	32.6	−0.12	0	17
		Fe—12% Si	30.1	−0.1	0	11
		Fe—26% Si	24.2	0.3	0	6

**Table 3:** Refined values of the hyperfine parameters of the two phases (bcc-(Fe,Si) and ThMn_12_-type structure) identified for Zr_0.4−x_Nd_x_Ce_0.6_Fe_10_Si_2_ (0 ≤ x ≤ 0.3) samples annealed at 1373 K for 72 h.

	Phase		B_hf_ (T) [±0.5]	δ (mm/s) [±0.01]	2ε (mm/s) [±0.01]	Area % [±2]
Zr_0.4_Ce_0.6_Fe_10_Si_2_						
	ThMn_12_	8i	24.4	−0.06	0.09	23
		8j	21.9	−0.1	0.09	26
		8f	19.8	−0.1	0.09	22
	bcc-(Fe,Si)	bcc-Fe	33.1	−0.12	−0.03	5
		Fe—4.5% Si	31.3	−0.08	−0.03	3
		Fe—12% Si	29.1	−0.16	−0.03	6
		Fe—26% Si	24.7	0.2	−0.03	5
Zr_0.3_Nd_0.1_Ce_0.6_Fe_10_Si_2_						
	ThMn_12_	8i	24.9	−0.1	0.06	20
		8j	22.5	−0.16	0.06	24
		8f	19.4	−0.22	0.06	18
	bcc-(Fe,Si)	bcc-Fe	33	−0.18	0.17	4
		Fe—4.5% Si	32.3	−0.08	0.17	2
		Fe—12% Si	30	−0.13	0.17	9
		Fe—26% Si	24	0.25	0.17	9
Zr_0.2_Nd_0.2_Ce_0.6_Fe_10_Si_2_						
	ThMn_12_	8i	25	−0.1	0.04	15
		8j	22.7	−0.15	0.04	21
		8f	19.5	−0.21	0.04	15
	bcc-(Fe,Si)	bcc-Fe	33	−0.03	0.06	6
		Fe—4.5% Si	32.3	−0.15	0.06	8
		Fe—12% Si	29.7	−0.12	0.06	10
		Fe—26% Si	24	0.27	0.06	10
Zr_0.1_Nd_0.3_Ce_0.6_Fe_10_Si_2_						
	ThMn_12_	8i	25.2	−0.12	−0.03	15
		8j	22.9	−0.14	−0.03	18
		8f	19.4	−0.24	−0.03	12
	bcc-(Fe,Si)	bcc-Fe	33.3	−0.06	−0.03	12
		Fe—4.5% Si	32.3	−0.13	−0.03	7
		Fe—12% Si	30	−0.07	−0.03	12
		Fe—26% Si	24	0.32	−0.03	9

## Data Availability

The data presented in this study are available on request from the corresponding author.
